# Acupuncture for Psychological Disorders Caused by Chronic Pain: A Review and Future Directions

**DOI:** 10.3389/fnins.2020.626497

**Published:** 2021-01-27

**Authors:** Lu-Lu Lin, Hong-Ping Li, Jing-Wen Yang, Xiao-Wan Hao, Shi-Yan Yan, Li-Qiong Wang, Fang-Ting Yu, Guang-Xia Shi, Cun-Zhi Liu

**Affiliations:** ^1^International Acupuncture and Moxibustion Innovation Institute, Beijing University of Chinese Medicine, Beijing, China; ^2^School of Acupuncture-Moxibustion and Tuina, Beijing University of Chinese Medicine, Beijing, China

**Keywords:** acupuncture, chronic pain, psychological states, review, clinical trials, biological mechanisms

## Abstract

Accumulating evidence supports an association between chronic pain and psychological disorders, a connection that seems to be bidirectional. Treating both the pain and psychological conditions together is essential for effective treatment outcomes. Acupuncture is a somatosensory-guided mind–body therapy that can tackle the multidimensional nature of pain with fewer or no serious adverse effects. In this review, we discuss the use of acupuncture in some conditions with a high incidence of psychological disorders caused by chronic pain: headache, musculoskeletal pain, low back pain, and cancer pain, focusing on the effect and potential mechanisms of acupuncture. Overall clinical studies indicated that acupuncture might effectively contribute to management of psychological disorders caused by chronic pain. Mechanistic studies showed that acupuncture significantly alleviated such psychological disorders by regulating the activity of amygdala and insula, and regulating functional connectivity of insular and limbic regions/medial prefrontal cortex in humans and the corresponding animal models. In addition, 5-HT in the dorsal raphe nucleus, opioid receptors in the cingulate cortex, and plasma met-enkephalin are involved in acupuncture relief of pain and psychological symptoms. Substantial evidences from animal and human research support a beneficial effect of acupuncture in psychological disorders caused by chronic pain.

## Introduction

Chronic pain is one of the most common conditions encountered by healthcare professionals. It is often defined as pain that persists more than 12 weeks, and may or may not be associated with an identifiable cause or actual tissue damage (Reid et al., 2015). Chronic pain is a worldwide problem and poses a significant economic burden on society. An estimated 20.4% of United States adults had chronic pain (Dahlhamer et al., 2018). In low-income and middle-income countries, the prevalence of chronic pain was 33% in adults (Jackson et al., 2015).

It has been proven that chronic pain contributed to psychological disorders (Kerns et al., 2011; Bliss et al., 2016). Psychological disorder, also called mental disorder, is a behavioral or mental pattern that causes significant distress or impairment of personal functioning (Stein et al., 2010). Depression and anxiety are the commonest psychological disorders caused by chronic pain (Canadian Mental Health Association, 2008). A study showed that, 15% of headache sufferers reported major depression, compared with 5% of non-sufferers (Kalaydjian and Merikangas, 2008). Another study indicated that cancer patients with pain, compared with those without pain, were more likely to report depression (Green et al., 2011). These findings in adults are similar to those in children. Psychosocial health and emotional functioning of children with migraine were adversely affected, compared with that of healthy children (Powers et al., 2003). Treating both the psychological disorders and pain together is essential for sustained treatment outcomes.

Acupuncture use dates back to over 3,000 years in China. In the past few decades, acupuncture is widely used to treat pain conditions all over the world. It is estimated that 8 million Americans have used acupuncture, predominantly for pain-related problems (Berman, 2007). However, studies of acupuncture for pain and psychological disorders are mostly carried out independently. Previous studies have shown that acupuncture might function as a somatosensory-guided mind–body therapy (Wang et al., 2007; Napadow et al., 2009) and could treat both pain and psychological diseases at once. In addition, recent advances in the effectiveness and the neurobiological pathways on acupuncture for chronic pain with psychological disorders have substantially increased people’s awareness about the therapeutic use of acupuncture.

This review summarizes the clinical data, mechanisms of action, and other information addressing the practice, rationale, and future directions of acupuncture for psychological disorders caused by chronic pain.

## Methods

The authors conducted a literature search of available sources describing issues relating to acupuncture, chronic pain, and psychological disorders. Research studies were selected based on research topics found in PubMed, Web of Science, and Embase from the database establishment up to the present time. We used the search string [pain(MeSH)] OR [headache(MeSH)] OR [osteoarthritis(MeSH)] OR [fibromyalgia(MeSH)] OR [cancer (MeSH)], AND [acupuncture(MeSH)], AND [psychological disorder(MeSH)], OR [mental disorder(MeSH)] OR [emotional (MeSH)], OR [anxiety(MeSH)], OR [depression(MeSH)] for PubMed and adapted it for Web of Science and Embase. The information provided in the selected studies was carefully evaluated and is described and discussed in the following sections.

## Clinical Evidence for Acupuncture

Clinical trials have documented the effectiveness of acupuncture on psychological disorders caused by chronic pain (Table 1). Among them, chronic headache, musculoskeletal pain, and cancer pain are the most common diseases. The majority of the studies suggest that acupuncture can significantly improve the emotional symptoms (such as anxiety and depression) in patients with chronic pain compared with both usual care and waiting list.

**TABLE 1:** Characteristics of studies on acupuncture for musculoskeletal pain, low back pain, and cancer pain.

Year	Journal	First author	Type of study	Diseases	Sample size (n)	Groups	Outcomes
2006	Headache	Melchart D	Epidemiological study	Headaches	2,022	Acupuncture	Depressive symptoms (ADS)
2018	Front Pharmacol	Jiang Y	Systematic Review, Meta-Analysis	Migraine	4,947	–	MSQ, SDS
2018	J Pain Res	Georgoudis G	RCT	Tension-type headache	44	Acupuncture and Stretching/Acupuncture, Stretching and Physiotherapy	HADS-A, HADS-D
2005	BMJ	Melchart D	RCT	Tension-type headache	270	Acupuncture/Sham acupuncture/Waiting list	ADS
2017	JAMA Intern Med	Zhao L	RCT	Migraine	249	Acupuncture/Sham acupuncture/Waiting list	MSQ, SAS, SDS
2005	Headache	Coeytaux RR	RCT	Headaches	74	Acupuncture and medical management/Medical management	Headache Impact Test, BDI
2006	Headache	Streng A	RCT	Migraine	114	Acupuncture/Metoprolol	ADS
2005	JAMA	Linde K	RCT	Migraine	302	Acupuncture/Sham acupuncture/Waiting list	ADS
2001	Cephalalgia	Karst M	RCT	Tension-type headache	69	Acupuncture/Sham acupuncture	Zerssen Depression Scale
2006	Rheumatology	Linde K	Observational study	Osteoarthritic pain	736	Acupuncture	CES-D
2020	Evid Based Complement Alternat Med	Di Carlo M	Open-Label Pragmatic Study	Fibromyalgia	96	Acupuncture	PCS
2005	Acupunct Med	He D	RCT	Chronic neck and shoulder pain	24	Electroacupuncture and ear acupressure/Sham acupuncture and sham ear acupressure	Frequency of tension and irritability, depression
2004	Pain	He D	RCT	Chronic neck and shoulder pain	24	Electroacupuncture and ear acupressure/Sham acupuncture and sham ear acupressure	Frequency of tension and irritability, depression
2002	Pain	Leibing E	RCT	Low back pain	131	Acupuncture and Physiotherapy/Sham and Physiotherapy/Physiotherapy	HADS
2013	Spine	Cho YJ	RCT	Low back pain	130	Acupuncture/Sham acupuncture	BDI
2008	Ind Health	Sawazaki K	Observational study	Low back pain	72	Acupuncture	POMS
2005	Lancet	Witt C	RCT	Knee osteoarthritis	294	Acupuncture/Sham acupuncture/Waiting list	ADS
2016	Eur J Integr Med	Lee GE	RCT	Shoulder pain	53	Acupuncture/Sham acupuncture	BDI
2006	Mayo Clin Proc	Martin DP	RCT	Fibromyalgia	50	Acupuncture/Sham acupuncture	FIQ, MPI
2007	Rheumatology	Williamson L	RCT	Knee osteoarthritis	181	Acupuncture/physiotherapy/standardized advice	HADS
2018	J Cancer	Lopez G	Retrospective analysis	Cancer pain	375	Acupuncture	PSS
2016	Support Care Cancer	Giron PS	RCT	Cancer pain	48	Acupuncture and kinesiotherapy/Kinesiotherapy	BDI
2017	J Pain Symptom Manage	Mehling WE	RCT	Cancer pain	138	Acupuncture and usual care/massage and usual care/usual care	POMS-SF

*ADS, Allgemeine Depressionsskala; MSQ, Migraine-Specific Quality of Life Questionnaire; SDS, Self-rating Depression Scale; RCT, Randomized Controlled Trial; HADS-A, the 7-item Hospital Anxiety and Depression Scale-anxiety; HADS-D, the 7-item Hospital Anxiety and Depression Scale-depression; SAS, Self-rating Anxiety Scale; BDI, the Beck’s Depression Inventory; CES-D, German version of the Center for Epidemiological Studies Depression Scale; PCS, Pain Catastrophizing Scale; POMS, the Profile of Mood States; FIQ, Fibromyalgia Impact Questionnaire; MPI, Multidisciplinary Pain Inventory; PSS, Psychological Distress; POMS-SF, Profile of Mood States Short Form.*

### Chronic Headache

Headaches are a common neurological complaint, typically accompanied by poor health-related quality of life (de Velasco et al., 2003). Tension-type headache is the most common type of headache (No authors listed., 2018). Meanwhile, migraine is one of the main causes of disability worldwide and medication overuse is the principal cause of chronic migraine (GBD 2016 Headache Collaborators, 2018). Studies have shown that 27% of patients with headache and 80% of patients with chronic migraine had depression (Jelinski et al., 2007; Evans, 2013). The prevalence of use of acupuncture and other complementary and alternative medicine approaches in chronic headaches was 62%, and the results were promising, particularly in patients with mood or anxiety disorders caused by headache (Nicholson et al., 2011; Kristoffersen et al., 2012). Clinical evidences showed that acupuncture could improve chronic headaches related symptoms, which led to improvements in emotional well-being of patients.

#### Effective for Both Pain and Psychological Disorder

A German epidemiological study that involved a total of 2,022 patients (732 with migraine, 791 with tension-type headaches, and 499 with other diagnoses) showed that the correlation was seen between average pain rating and depression. Meanwhile, headache patients reported significant and clinically relevant improvements in headache frequency, pain intensity, and depression level (measured with the Allgemeine Depressionsskala scale) at the end of the acupuncture treatment of 6–15 sessions (each session lasted 30 min) compared with baseline (Melchart et al., 2006).

For migraine, a meta-analysis in 2018, which involved 61 trials, showed that acupuncture was superior to sham acupuncture in reducing pain intensity (measured with the Visual Analog Scale) and enhancing the quality of life and psychological disorders (measured with Migraine-Specific Quality of Life Questionnaire) of patients (Jiang et al., 2018).

In a study, 44 participants with tension-type headache received acupuncture add stretching or acupuncture, stretching add physiotherapy during a 4-week intervention period including 10 treatment sessions, each session lasted 20 min. After treatment, significant improvements in pain intensity (measured with the Visual Analog Scale), anxiety, depression (measured with the Hospital Anxiety and Depression Scale), and pain catastrophizing (measured with the Pain Catastrophizing Scale) levels were noted for both groups in the study (Georgoudis et al., 2018). These findings were in accordance with those of Melchart’s study (Melchart et al., 2005). In Melchart’s study, 270 participants with tension-type headache were randomly assigned to acupuncture (12 sessions of 30 min over 8 weeks), sham acupuncture (superficial needling at non-acupuncture points), or waiting-list group. At the end of the treatment, compared with the waiting-list group, participants receiving acupuncture fared significantly better for the headache days and depression (measured with Allgemeine Depressionsskala). However, acupuncture was not significantly more effective than sham acupuncture.

A randomized clinical trial (RCT) of 249 participants with migraine compared electroacupuncture (five sessions per week for 4 weeks, each session lasted 30 min) with sham acupuncture (insertion of sterile disposable needles at non-acupuncture points) or waiting-list treatment and found that electroacupuncture produced a greater reduction in the frequency of migraine and a better therapeutic effect on emotional functional subscale of Migraine-Specific Quality of Life Questionnaire than sham acupuncture or waiting-list treatment (acupuncture vs. sham acupuncture, *P* = 0.01; acupuncture vs. waiting-list, *P* < 0.01). However, for Self-rating Depression Scale, there were no significant differences among the three groups (Zhao et al., 2017).

#### Effective for Pain but Ineffective for Psychological Disorder

An RCT of 74 participants with chronic daily headache compared medical management plus 10 acupuncture sessions to medical management during 6 weeks; each acupuncture session lasted 30 min. It found that, medical management plus acupuncture was associated with a decrease in 3.0 points (CI, 1.0–4.9) on the headache burden (measured with Headache Impact Test) relative to medical management without acupuncture at the 6-week follow-up. However, the depression level (measured with the Beck Depression Inventory scores) did not improve significantly after acupuncture treatment (Coeytaux et al., 2005). These findings are consistent with those of another RCT of 114 participants with migraine, in which the effect of acupuncture (8–15 sessions of 20–30 min over 12 weeks) or metoprolol was studied on average pain, days with migraine, and depression (measured with Allgemeine Depressionsskala) (Streng et al., 2006). The results showed that acupuncture participants fared significantly better for average pain (*P* = 0.005). However, for days with migraine and Allgemeine Depressionsskala, there was no significant difference between the acupuncture and metoprolol groups (*P* > 0.05).

In Linde’s study, 302 participants with migraine were randomly assigned to acupuncture (12 sessions for 30 min over 8 weeks), sham acupuncture (superficial needling at non-acupuncture points), or waiting-list group (Linde et al., 2005). At the end of 12 weeks, acupuncture was significantly better than the waiting-list in the intensity of headache (*P* < 0.001), but there was no significant difference between acupuncture and sham acupuncture groups. In addition, the depression level (measured with the Allgemeine Depressionsskala depression scale) did not improve significantly after acupuncture treatment. No difference was detected among the three groups.

#### Ineffective for Either Pain or Psychological Disorder

In an RCT, 69 participants with tension-type headache were randomly assigned to acupuncture (two sessions per week for 5 weeks, each session lasting 30 min) or sham acupuncture group (blunt needles on non-acupuncture points without penetration). No significant differences between the two groups with respect to visual analog scale and frequency of headache attacks, and von Zerssen Depression Scale could be observed after treatment (Karst et al., 2001).

#### Summary

In summary, the available evidence indicates that depression should have the most concern in all psychological disorders in the clinical studies of headache. In addition, anxiety and pain catastrophizing has also been studied. Most studies supported acupuncture for the treatment of psychological disorders caused by headache, despite true acupuncture interventions did not seem to be superior to sham acupuncture in some studies. The specific effects of acupuncture, relative to appropriate control groups, have yet to be tested in methodologically sound clinical trials.

### Musculoskeletal Pain

Musculoskeletal conditions are the highest contributor to global disability (GBD 2017 Disease and Injury Incidence and Prevalence Collaborators, 2018). They are typically characterized by pain and restricted mobility, reducing people’s ability to work and participate in social roles with associated impacts on mental well-being (Sleijser-Koehorst et al., 2019; World Health Organization, 2019). In addition, psychological disorders, such as depression and anxiety can increase the incidence risk of musculoskeletal diseases (Gaspersz et al., 2018; Burston et al., 2019). Effectiveness of acupuncture in the treatment of musculoskeletal diseases has been reviewed in detail elsewhere (MacPherson et al., 2017). In this section, we focus on psychological disorders caused by several common chronic pain conditions (osteoarthritis, fibromyalgia, chronic neck and shoulder pain, and low back pain) (World Health Organization, 2019) in acupuncture clinical practice.

#### Effective for Both Pain and Psychological Disorder

In a German observational study, 736 patients with hip or knee osteoarthritis were treated with 6–15 sessions of acupuncture. Duration of sessions had to be at least 30 min. After treatment, patients reported less pain (measured with the pain subscale of Western Ontario and McMaster Universities Osteoarthritis Index) and lower depression scores (measured with Allgemeine Depressionsskala). All changes were statistically highly significant (*P* < 0.001) (Linde et al., 2006).

A pragmatic study, which involved 96 patients with fibromyalgia, showed that significant changes in pain (measured with the Fibromyalgia Impact Questionnaire) and negative psychological perceptions——pain catastrophizing (measured with the Pain Catastrophizing Scale) have been documented at the end of acupuncture treatment (once a week for 8 weeks). Each session lasted 30 min (Di Carlo et al., 2020).

In an RCT with 6 months and 3 years follow-ups, 24 patients with chronic neck and shoulder pain compared electroacupuncture add ear acupressure for 45 min against sham acupuncture (insertion of sterile disposable needles at non-acupuncture points) add ear acupressure at the non-acupoints for the same duration. Electroacupuncture was applied for 10 sessions during 3–4 weeks. After treatment, compared with the sham acupuncture group, electroacupuncture significantly improved the pain threshold in the neck and shoulder regions, anxiety, depression, and satisfaction with life (*P* < 0.05). At 6 months and 3 years follow-ups the electroacupuncture group showed further improvements in these variables and was again significantly different from the sham acupuncture group (He et al., 2005). These results were congruent with the findings of the author’s other study, which reported that adequate acupuncture treatment reduced chronic pain in the neck and shoulders, and the effect lasted for 3 years (He et al., 2004).

In Leibing’s study, 131 patients with low back pain were treated with acupuncture (20 sessions for 30 min over 12 weeks) plus physiotherapy (26 sessions for 30 min over 12 weeks), sham acupuncture (superficial needling at non-acupuncture points) plus physiotherapy, and physiotherapy alone over 12 weeks. It reported improved pain intensity (measured with Visual Analog Scale) and psychological stress (measured with Hospital Anxiety and Depression Scale) in the acupuncture group (acupuncture vs. sham acupuncture, *P* < 0.05; acupuncture vs. physiotherapy group, *P* < 0.05) (Leibing et al., 2002).

In an RCT, 130 patients with low back pain were randomized into receiving acupuncture versus sham acupuncture (semi-blunt needles on non-acupuncture points without penetration) twice a week for 6 weeks. Each session lasted 15–20 min. It found that acupuncture reduced pain intensity of chronic low back pain (measured with Visual Analog Scale) better than sham acupuncture (*P* < 0.05). In addition, depression level (measured with Beck Depression Inventory) of the acupuncture group was improved during the entire study (*P* < 0.01), but no significant effect was observed between the groups (Cho et al., 2013).

An intervention study of 72 patients with chronic low back pain found that after 8 weeks of acupuncture treatment (once a week, each treatment lasted 15 min), patients reported a significant decrease in the pain (measured with Numerical Rating Scale) and in the total mood disturbance score, as measured by the Profile of Mood States (*P* < 0.05). However, tension-anxiety of the Profile of Mood States did not show significant changes (Sawazaki et al., 2008).

#### Effective for Pain but Ineffective for Psychological Disorder

An RCT of 294 patients with knee osteoarthritis published in Lancet suggested that acupuncture treatment improved knee pain (measured with the pain subscale of Western Ontario and McMaster Universities Osteoarthritis Index) compared with sham acupuncture (superficial needling at non-acupuncture points) and waiting list at the end of 8 weeks (*P* < 0.001). Both the acupuncture and sham acupuncture treatments consisted of 12 sessions of 30 -min duration. However, acupuncture did not reduce the depression (measured with Allgemeine Depressionsskala) of patients compared with neither waiting-list nor sham acupuncture (Witt et al., 2005).

In an RCT, 53 participants with shoulder pain were randomly allocated into either the acupuncture or sham acupuncture (non-acupuncture point shallow penetrating acupuncture) group. Acupuncture was offered to each participant three sessions per week for 3 weeks; each session lasted 15 min. For pain level (measured with Visual Analog Scale), the acupuncture group demonstrated a significant change compared with the sham acupuncture group at the third week of treatment. However, there were no significant differences between the two groups on the Beck Depression Inventory (Lee et al., 2016).

#### Effective for Psychological Disorder but Ineffective for Pain

In an RCT, 50 patients with fibromyalgia were randomly assigned to acupuncture (every 2– 4 days during 2 weeks for six sessions, each session lasted 20 min) or sham acupuncture group (the tip of the needle is blunt without puncturing the skin). After 1 month treatment, anxiety, as measured by the Fibromyalgia Impact Questionnaire and Multidisciplinary Pain Inventory, was significantly improved in the acupuncture group compared with the sham acupuncture group (*P* < 0.05). However, there was no significant difference between the two groups for pain and depression (measured with the Fibromyalgia Impact Questionnaire) after treatment (Martin et al., 2006).

#### Ineffective for Either Pain or Psychological Disorder

An RCT comparing acupuncture (one sessions per week for 6 weeks, each session lasted 20 min) with physiotherapy and standardized advice in 181 patients with severe osteoarthritic knee pain awaiting knee arthroplasty found no significant changes in knee pain (measured with the pain subscale of Western Ontario and McMaster Universities Osteoarthritis Index), anxiety, and depression (measured with Hospital Anxiety and Depression Scale) among the three groups (Williamson et al., 2007).

#### Summary

Similar to chronic headache, the most concerned psychological disorders are anxiety and depression in studies of musculoskeletal pain. In some studies, the Pain Catastrophizing Scale and the Profile of Mood States were used to assess the psychological disorders in patients with musculoskeletal pain. In most cases, acupuncture is effective for both pain and psychological disorders. Only in a few cases, acupuncture is ineffective for musculoskeletal pain. It may be related to the severity of the disease and the dose of acupuncture.

### Cancer Pain

Cancer is among the leading causes of death worldwide. In 2016, there were an estimated 15.5 million cancer survivors in the United States (National Cancer Institute, 2018). Evidences showed that a third of cancer survivors suffered from pain. Moreover, cancer pain was significantly associated with the prevalence of depression, feeling worried, nervous, or anxious (Sanford et al., 2019). On the basis of several studies highlighted in this section, acupuncture may improve psychological symptoms caused by chronic cancer pain including tension, anxiety, and depression.

#### Effective for Both Pain and Psychological Disorders

In a retrospective analysis, 280 cancer pain patients received personalized acupuncture treatments one or two sessions weekly for 3–4 weeks (each session lasted 20–30 min). It found that acupuncture showed a significant reduction in numbness/tingling, anxiety, depression, and other psychological disorders (measured with Edmonton Symptom Assessment Scale) at the end of the treatment (*P* < 0.001) (Lopez et al., 2018). These findings are consistent with those of an RCT of 48 breast cancer patients. Patients in the study were randomly assigned to acupuncture (one session per week for 10 weeks) plus kinesiotherapy or kinesiotherapy group (Giron et al., 2016). Kinesiotherapy group treated with a predefined kinesiotherapy protocol of 30 min; acupuncture plus kinesiotherapy group treated with the same kinesiotherapy group protocol followed by another 30 min of acupuncture. At the end of the treatment, both groups showed statistically significant improvement of the pain and depression (measured with the Beck Depression Inventory). There was no difference between groups.

An RCT, 138 postoperative cancer patients, compared acupuncture (one session for 20 min), massage (one session for 10–30 min) add usual care against usual care alone. After treatment (postoperative Days 1–3), massage and acupuncture add usual care significantly decreased perioperative symptoms, such as pain and depressive mood (measured with Profile of Mood States Short Form) when compared with usual care alone (each *P* < 0.05). Tension/anxiety improved in the acupuncture group on postoperative Day 1 more than in the usual care group (*P* = 0.048), but these improvements were not maintained on postoperative Day 2 and postoperative Day 3 (Mehling et al., 2007).

#### Summary

In these studies of cancer pain, anxiety, and depression are the psychological disorders of concern. Studies have found statistically significant improvements in pain intensity with acupuncture. Acupuncture applied to chronic cancer pain conditions is also typically beneficial with respect to anxiety and depression.

## Mechanisms of Acupuncture

### The Expression of Proteins in the Amygdala

Acupuncture may relieve psychological disorders caused by chronic pain through altering the expression of proteins in the amygdala. Amygdala is a group of closely associated nuclei situated deep in the temporal lobe (Goddard, 1964). It has long been known that the amygdala has been associated with a range of cognitive functions, especially emotion (Baxter and Murray, 2002; Salzman and Fusi, 2010; Kirkby et al., 2018). A recent study has shown that the central nucleus of the amygdala neurons selectively influence pain by blocking nociception and have widespread inhibitory projections to many emotional pain-processing centers (Hua et al., 2020). Moreover, the central nucleus of the amygdala is an important part of the neural circuit mechanisms underlying comorbid depressive symptoms in chronic pain (Zhou et al., 2019). A study in people with anxiety caused by migraine showed that compared with the healthy people, migraine patients exhibited a larger volume of the amygdala. Furthermore, there was a significantly positive association between the gray matter volume of the amygdala and anxiety (Liu et al., 2017). Consistent with these results, carpal tunnel syndrome patients responded to acupuncture with greater deactivation in the amygdala compared to healthy people (Napadow et al., 2007). A preclinical investigation indicated that electroacupuncture inhibited the complete Freund’s adjuvant-induced emotional response in the conditioned place aversion test by down- or upregulating the protein expression in the amygdala of pain aversion rats (Wu et al., 2019).

### Insular, Functional Connectivity of Insular and Limbic Regions/Medial Prefrontal Cortex

Acupuncture could relieve psychological disorders caused by chronic pain through regulating insular itself, and functional connectivity of insular and limbic regions/medial prefrontal cortex. The insula, also known as the insular cortex, is bilaterally located deep within the lateral sulcus or the fissure. The anterior insular cortex has reciprocal connections to the limbic regions such as the anterior cingulate cortex, the prefrontal cortex, and the amygdala. Thus, it has been implicated in limbic function, such as emotional feelings and pain (Namkung et al., 2017). A study indicated that negative emotional stimuli increased the activation in the anterior insula and was relevant to pain intensity (Stancak and Fallon, 2013). Another study showed that pain increased the anti-correlation between the anterior insula and posterior cingulate cortex (Cottam et al., 2018). Furthermore, clinical anxiety, depression, and pain intensity were positively correlated with increased connectivity between the anterior insula and medial prefrontal cortex (As-Sanie et al., 2016). A functional imaging study in healthy humans has shown that acupuncture at PC6 (Neiguan) increased the activity in the insula and medial prefrontal cortex, and decreased the activity in the posterior cingulate (Napadow et al., 2009). Moreover, in a study of the mechanism of acupuncture in fibromyalgia patients, the authors found that after acupuncture, a complex interaction might occur between the insula cortex and the anterior cingulate, which induced a relief of pain and improvement of emotional experience (de Tommaso et al., 2014).

### The 5-HT of the Dorsal Raphe Nucleus

It has been proven that 5-HT in the dorsal raphe nucleus of pain-related depression condition was increased through acupuncture. The dorsal raphe nucleus is located on the midline of the brainstem. It is a major source of neuromodulators in the central nervous system and is the largest of the serotonergic nuclei (Huang et al., 2019). Changes in 5-HT neuron function in the dorsal raphe nucleus have been implicated in various neuropsychiatric diseases, such as major depressive disorder and bipolar disorder (Mahmood and Silverstone, 2001; Vaswani et al., 2003; McDevitt et al., 2011). In people with depression, the dorsal raphe nucleus may be decreased in size and in serotonin transporter (Arango et al., 2001; Matthews and Harrison, 2012). In addition, the serotonergic system in the dorsal raphe nucleus has been proved to participate in the descending modulation of pain (Freitas et al., 2008; Ossipov et al., 2010) and has a role in the effects of acupuncture on emotional disorders caused by pain (Yang et al., 2017). A study in reserpine-induced pain-depression dyad rat model showed that electroacupuncture of 100 Hz alleviated the pain-depression dyad and upregulated 5-HT in the dorsal raphe nucleus of reserpine-injected rats. Intracerebroventricular injection of 5-HT resynthesis inhibitor suppressed the upregulation of 5-HT in the dorsal raphe nucleus by 100 Hz of electroacupuncture and partially counteracted the analgesic and antidepressive effects of 100 Hz of electroacupuncture (Wu et al., 2017).

### The Opioid Receptors in the Cingulate Cortex

Acupuncture can improve the emotion of pain by modulating the opioid receptors in the cingulate cortex. The cingulate cortex situates in the medial aspect of the cerebral cortex and is an integral part of the limbic lobe (Vogt, 2005). Recent evidence suggests that the cingulate cortex is a central hub in the pain matrix and is highly connected with most other brain areas involved in the processing of pain (Bliss et al., 2016). Meanwhile, the anterior cingulate cortex is a critical hub for emotion disorders caused by pain, such as depression (Barthas et al., 2015). In a study of the electroacupuncture effect in a complete Freund’s adjuvant-induced inflammatory hyperalgesia rat model, authors found that 10 Hz of electroacupuncture at GB30 significantly inhibited complete Freund’s adjuvant-induced place aversion assessed through conditioned place avoidance, indicating that electroacupuncture inhibited the emotional response. Intra-rostral anterior cingulate cortex administration of antagonists against opioid receptor antagonist blocked electroacupuncture action, which demonstrated that electroacupuncture activated opioid receptors in the rostral anterior cingulate cortex to inhibit the emotion of pain (Zhang et al., 2011).

### Plasma Met-Enkephalin

Plasma met-enkephalin is involved in acupuncture relief of pain and psychiatric symptoms. A study in patients with chronic pain and serious psychiatric difficulties indicated that acupuncture treatment was associated with significant improvement in both pain and depression, and the degree of symptom relief was correlated with the degree of post-acupuncture increase in plasma met-enkephalin immunoreactivity. However, acupuncture did not change the level of plasma β-endorphin concentrations. In addition, patients with a lower baseline plasma met-enkephalin concentration were more likely to get successful acupuncture pain relief (Kiser et al., 1983).

## Summary and Future Directions

Overall, the results indicate that studies of the use of acupuncture to promote psychological states in patients with chronic pain show promising results, although high-quality and large sample size studies are needed. At present, psychological disorders caused by chronic pain have been proven to be associated with anterior cingulate cortex hyperactivity and long-term potentiation (Bliss et al., 2016; Sellmeijer et al., 2018); functional impairment in noradrenergic circuits associated with locus coeruleus and prefrontal cortex (Alba-Delgado et al., 2013); increased glutamate level at the hippocampus (Fasick et al., 2015); the dysfunction of the mesolimbic dopamine system (Finan and Smith, 2013; Serafini et al., 2020); the dysfunction of the serotoninergic system in the dorsal raphe nucleus (Zhou et al., 2019); the dysfunction of opioid receptors in the amygdala (Narita et al., 2006) and the nucleus accumbens and its interactions with the medial prefrontal cortex and midbrain dopaminergic neurons (Benarroch, 2016). Several potential mechanisms of acupuncture for psychological disorders caused by chronic pain have been suggested, such as regulating the activity of amygdala and insula. Furthermore, functional connectivity of insular and limbic regions/medial prefrontal cortex was regulated by acupuncture. In addition, 5-HT in the dorsal raphe nucleus, opioid receptors in the cingulate cortex, and plasma met-enkephalin were involved in acupuncture relief of psychological symptoms caused by chronic pain.

Even if true acupuncture interventions do not seem to be superior to sham acupuncture (Karst et al., 2001; Linde et al., 2005; Melchart et al., 2005; Witt et al., 2005; Cho et al., 2013), in some cases, we cannot ignore the effect of acupuncture. This is because sham acupuncture (even if non-penetrating needles) may have direct physiological effects (Lund and Lundeberg, 2006; Napadow et al., 2009; Liu et al., 2017), and acupuncture may have a significant placebo effect, especially in studies with continuous subjective outcomes. Acupuncture involves repeated sessions, which do intense acupuncturist contact. If there is a good patient–therapist relationship, these regular visits may reduce anxiety, acquire social support, and improve well-being (Feine and Lund, 1997). However, acupuncture is more than a placebo. The total effects of acupuncture include the specific effects related to correct needle insertion according to acupuncture theory, non-specific physiologic effects of needling, and non-specific psychological effects (Vickers et al., 2012). Additionally, evidence showed that placebo effect was small and probably not at all of any clinical relevance in terms of long-term effects (Carlsson, 2002). In contrast, the effect of acupuncture for patients with psychological disorders caused by chronic pain can persist from 3 months to 3 years (He et al., 2004, 2005; Hansson et al., 2007; MacPherson et al., 2017).

It should be noted that acupuncture cannot improve emotional symptoms caused by chronic pain in some cases. We consider several possible explanations. First, it may be related to the severity of disease. Generally, a less depressed patient is more likely to respond to any therapy for chronic pain (Lewith and Kenyon, 1984), and a patient with severe pain is less likely to respond to acupuncture in psychological states (Williamson et al., 2007). Second, it may be related to individual differences. A study that separated the patients with chronic pain into responders or non-responders, found that the responder group had an average pain reduction following acupuncture of 74%; instead, the non-responder group actually reported an average 2% more pain following acupuncture. Other outcomes were similar to these results (Toomey et al., 1977). Another study showed that the baseline gray matter volume of the medial prefrontal cortex was significantly associated with the improvement in migraine symptoms after sham acupuncture treatment. In other words, individual differences of the baseline brain structure in the pain modulatory system served as a substrate in predicting the prognosis of acupuncture treatment in migraineurs (Liu et al., 2017). Third, it may be related to sex. The prevalence rates of pain and psychological disorders were higher in women than in men. Furthermore, women are more sensitive to acupuncture treatment. A study found that the prevalence of depression/anxiety was 27 and 14% between women and men; for pain, 24 and 19%, respectively (Narusyte et al., 2020). Another study assessed the effects of acupuncture on mood expressions in patients with chronic pain. It showed that for women, positive mood scales highly significantly increased and negative mood scales decreased after acupuncture treatment in their intensity highly. However, there was no significant influence of acupuncture on positive or negative mood scales of the male patients (Acker et al., 2015).

Demand for mind–body therapy approaches in the treatment of psychological disorders caused by chronic pain is high, and their use is increasing. It is generally known that acupuncture is a time-honored form of pain relief. The effect and mechanisms of acupuncture on persistent pain have been widely studied (Zhang et al., 2014). Accumulated evidences indicate that acupuncture can also function as a somatosensory-guided mind–body therapy to treat psychological disorders caused by chronic pain (Napadow et al., 2009). Although there is an increased use of acupuncture in clinical practice, due to the shortcomings of current research, these data remain inconclusive about its effectiveness in the management of psychological disorders caused by chronic pain. First, in RCTs, the psychological states assessment is usually used as secondary outcomes and has not received enough attention. In addition, most studies have not directly compared the connection between pain relief and psychological states. Second, the sample size of most RCTs is small, which is associated with an overestimation of treatment effects. Further, the scientific merits of some studies are often limited owing to the poor study design, with non-randomized controls. Third, although many nuclei have been found to be involved in acupuncture inhibition of psychological disorders caused by chronic pain, it is not clear how they work together.

In the future, large randomized trials and well-designed studies would be needed to investigate the effect of acupuncture on psychological states in people with chronic pain. In order to be effective, the comprehensive assessment-treatment of recalcitrant chronic pain and related psychological states is needed. More reasonable sham interventions also need to be designed to minimize the placebo effect of acupuncture. Additionally, the role of the genetic factors and sex are also an especially promising new area of research that should provide even greater insights into etiological mechanisms of psychological disorders caused by chronic pain that may account for important individual differences in the pain and emotion experience and one’s response to it. Finally, how the emotion/pain-related brain nuclei work together and the peripheral mechanism of acupuncture on chronic pain-related psychological states should be further studied.

## Conclusion

In chronic pain conditions (headache, osteoarthritis, fibromyalgia, chronic neck and shoulder pain, low back pain, and cancer pain), acupuncture may improve pain and psychological disorders. Acupuncture may mediate its anti-pain, anti-anxiety, anti-depression, and other therapeutic effects via regulating brain regions and their related substances. These brain regions play a central role in pain as well as in the regulation and integration of emotion and sensorimotor functions. However, the effect of acupuncture remains to be assessed in large and well-designed clinical trials. Further, there is a clear need for further studies to investigate the peripheral mechanism of acupuncture on chronic pain-related psychological states.

## Author Contributions

L-LL, G-XS, H-PL, J-WY, and X-WH wrote the manuscript. L-QW, F-TY, and L-LL elaborated the tables. G-XS, S-YY, and C-ZL participated in the conception of the idea and revised the manuscript. All the authors contributed to the article and approved the submitted version.

## Conflict of Interest

The authors declare that the research was conducted in the absence of any commercial or financial relationships that could be construed as a potential conflict of interest.
